# Omicron BA.2.75 Subvariant of SARS-CoV-2 Is Expected to Have the Greatest Infectivity Compared with the Competing BA.2 and BA.5, Due to Most Negative Gibbs Energy of Binding

**DOI:** 10.3390/biotech11040045

**Published:** 2022-10-11

**Authors:** Marko Popovic

**Affiliations:** School of Life Sciences, Technical University of Munich, 85354 Freising, Germany; marko.popovic@tum.de

**Keywords:** BA.2.75 variant, Gibbs energy of binding, binding rate, infectivity, SARS-CoV-2, phenomenological equation, nonequilibrium thermodynamics, chemical kinetics, Omicron

## Abstract

Omicron BA.2.75 may become the next globally dominant strain of COVID-19 in 2022. The BA.2.75 sub-variant has acquired more mutations (9) in spike protein and other genes of SARS-CoV-2 than any other variant. Thus, its chemical composition and thermodynamic properties have changed compared with earlier variants. In this paper, the Gibbs energy of the binding and antigen-receptor binding rate was reported for the BA.2.75 variant. Gibbs energy of the binding of the Omicron BA.2.75 variant is more negative than that of the competing variants BA.2 and BA.5.

## 1. Introduction

Multicellular organisms can be considered as open thermodynamic systems exhibiting growth [1,2,3]. Microorganisms, including viruses, represent open thermodynamic systems with the property of growth through multiplication [4,5,6,7,8,9,10,11]. Microorganisms perform chemical, physical, and biological interactions with their environment, other microorganisms, and their host [4,5,7,12,13,14,15,16]. The basic condition for virus–host interaction is the presence of an appropriate antigen on the virus and an appropriate receptor on the host cell [12]. The receptor on human cells susceptible to SARS-CoV-2 is angiotensin-converting enzyme 2 (ACE2). SARS-CoV-2 binds to its host cells using its antigen, the spike glycoprotein trimer (SGP) [17,18]. 

Microorganisms represent open thermodynamic systems, exchanging matter and energy with their surroundings [2]. Thermodynamic properties are available for more than 50 microorganisms [7]. Thermodynamic analysis has been carried out for biochemical processes performed by microorganisms [19,20,21,22,23,24]. The thermodynamic driving force for the growth of microorganisms was analyzed by von Stockar [5,6,25]. The applications of the laws of thermodynamics in biology and medicine are available in the literature [26,27,28,29,30,31,32]. Hansen underlined the importance of calorimetry in life sciences and drew a parallel between biological evolution and the laws of thermodynamics [33,34,35]. The relationship between evolution and the laws of thermodynamics was been discussed by Bejan and Lorente [36]. The constructal law was used to compare evolution through time of animate and inanimate systems [36]. It was found that same patterns can be observed in evolution of systems ranging from living organisms, through rivers to technology [36,37,38]. In addition, the relationship between information, entropy, and self-assembly in living organisms was analyzed in refs. [39,40]. Skene [41] developed a thermodynamic model of biological evolution, combining diversification, post-extinction recovery, and the likelihood of discovery of the fossil record. Morowitz discussed the role of thermodynamics in life processes [42,43] and the origin of life [44]. 

As of 2022, data have been published on thermodynamic properties on Monkeypox and Vaccinia viruses [45]. The atom-counting method was developed to obtain the empirical formulae and thermodynamic properties of viruses [46].

SARS-CoV-2 is an RNA virus. RNA viruses mutate more often than DNA viruses [47]. Starting from the original Hu-1 variant, SARS-CoV-2 has developed several dozen mutations [48,49]. These mutations have contributed to an increase in infectivity, in accordance with the predictions of theory of evolution [50]. Some mutations have contributed to an increase in infectivity, while others to immune response evasion [18,48]. 

The goal of this paper was to, based on the available literature data, calculate the value of the standard Gibbs energy of binding of the BA.2.75 variant, as well as to determine the antigen–receptor binding rate. Moreover, using a mechanistic model, an explanation was provided for the increase in infectivity of BA.2.75 compared with BA.2 and BA.5, which competed during the summer wave of COVID-19, in July and August 2022. 

## 2. Materials and Methods

Dissociation equilibrium constants for the spike glycoprotein trimer (SGP) of SARS-CoV-2 to the human angiotensin-converting enzyme 2 (ACE2) were taken from Cao et al. [51]. Their values are presented in Table 1. Their measurements were taken at 25 °C by surface-plasmon resonance [51].

### 2.1. Gibbs Energy of Binding and Dissociation Equilibrium Constant 

The binding of the virus antigen (SGP) to the host-cell receptor (ACE2) represents a chemical reaction. The rate of this chemical reaction can be calculated using the binding phenomenological equation

(1) $$r_{B}=-\frac{L_{B}}{T}{∆}_{B}G$$

where *r_B_* is the rate of binding of SGP to hACE2, *L_B_* the binding phenomenological coefficient, *T* temperature and Δ*_B_G* the Gibbs energy of binding of SGP to hACE2 [4,52,53,54,55]. The binding phenomenological equation shows that the rate of binding is proportional to the negative value of the Gibbs energy of binding. 

Barton et al. [56] reported that mutations in viruses lead to changes in binding affinity and standard Gibbs energy of binding. The standard Gibbs energy of binding quantifies the strength with which the virus antigen binds to host-cell receptor. The strength of antigen–receptor interactions is related to the ability of coronaviruses to infect human hosts [57]. Mutations induce significant changes in SGP conformation [58]. The mutations that lead to higher binding affinity are promoted by evolution through natural selection [58]. The quantitative measure of binding affinity is the Gibbs energy of binding [13,14,15,16]. 

The Gibbs energy of binding can be determined using dissociation equilibrium constants. The standard Gibbs energy of binding, Δ*_B_G*⁰, is given by the equation

(2) $${∆}_{B}G^{0}=-R_{g}T\ln\left(K_{B}\right)$$

where *R_g_* is the universal gas constant, *T* the temperature, and *K_B_* the binding equilibrium constant [52,59]. *K_B_* can be found as the reciprocal of the dissociation equilibrium constant, *K_D_* [59].

(3) $$K_{B}=\frac{1}{K_{D}}$$

The binding and dissociation equilibrium constants are defined for the antigen–receptor binding reaction.

(4) A+R⇄AR

where *A* represents the virus antigen (SGP), *R* the host-cell receptor (hACE2), and *AR* the antigen–receptor complex [52,59]. Thus, *K_D_* is defined through the free antigen concentration [*A*], free receptor concentration [*R*], and antigen–receptor complex concentration [*AR*] [52,59]

(5) $$K_{D}=\frac{{\left[A\right]}^{eq}{\left[R\right]}^{eq}}{{\left[AR\right]}^{eq}}$$

where the superscript “*eq*” was added to denote that the concentrations are at chemical equilibrium. 

The Gibbs energy of binding was calculated from the binding equilibrium constant, which in turn was found from the dissociation equilibrium constant. Δ*_B_G*⁰ is the thermodynamic driving force for the chemical reaction of antigen–receptor binding.

### 2.2. The kinetic Method and Rate Constants 

The chemical reaction of antigen–receptor binding is reversible. It consists of forward and backward half-reactions. The forward half-reaction is *A* + *R* → *AR*, where the free virus antigen and host cell receptor bind to form the antigen–receptor complex. Thus, it is of the second order. The rate of the forward half-reaction, *r_on_*, is given by the law of mass action [60,61], depending on the concentration of the free antigen, [*A*], and the free receptor [*R*].

(6) $$r_{on}=k_{on}\left[A\right]\left[R\right]$$

where *k_on_* is the rate constant of the forward half-reaction, which is also known as the on-rate constant or association rate constant [54,59]. On the other hand, the backward half-reaction is *AR* → *A* + *R*, where the antigen receptor complex dissociates into free antigen and receptor. The rate of the backward half-reaction, *r_off_*, is

(7) $$r_{off}=k_{off}\left[AR\right]$$

where *k_off_* is the rate constant for the backward half-reaction, which is also known as the off-rate constant [54,59]. The rates of the forward and backward half-reactions are combined to find the overall binding rate, *r_B_*, through the equation [54]

(8) $$r_{B}=r_{on}-r_{off}$$

At equilibrium, the *r_B_* becomes zero. This means that at equilibrium, the rates of forward, *r_on_^eq^*, and backward, *r_off_^eq^*, half-reactions are equal [55,60,61].

(9) $$r_{on}^{eq}=r_{off}^{eq}$$

### 2.3. Binding Phenomenological Coefficient

The abovementioned consideration presents the perspective of chemical kinetics on antigen–receptor binding. However, the antigen–receptor binding rate can also be calculated using the binding phenomenological Equation (1). This requires knowledge of the binding phenomenological coefficient, *L_B_*. The binding phenomenological coefficient can be calculated using the equation [55]

(10) $$L_{B}=\frac{r_{on}^{eq}}{R_{g}}$$

This equation can be combined with Equation (6) to obtain [54]

(11) $$L_{B}=\frac{k_{on}{\left[A\right]}^{eq}{\left[R\right]}^{eq}}{R_{g}}$$

Combining with Equation (5) results in [54]

(12) $$L_{B}=\frac{k_{on}K_{D}{\left[AR\right]}^{eq}}{R_{g}}$$

The value of *K_D_* for SARS-CoV-2 variants is very small, on the order of nM. Thus, the chemical equilibrium of antigen–receptor binding is shifted towards the antigen–receptor complex. This means that the majority of virus particles will be attached to host cells. This implies that the equilibrium concentration of the antigen receptor complex is approximately equal to the total concentration of virus particles in the organism: [*AR*]*^eq^* ≈ [*V*]*_tot_* [54]. Therefore, the equation above is transformed into [54]

(13) $$L_{B}=\frac{k_{on}K_{D}{\left[V\right]}_{tot}}{R_{g}}$$

Sender et al. [62,63] found that the value of [*V*]*_tot_* is 1 × 10^7^ RNA copies per gram of tissue. A SARS-CoV-2 virus particle contains a single copy of its RNA genome [64,65,66]. This means that the concentration of virus particles is 1 × 10^7^ RNA copies per gram of tissue [54]. This is combined with the density of human tissues, which is 1050 g/dm^3^ [67], resulting in a total concentration of virions of 1.74 × 10^−14^ M [54]. This result is substituted into Equation (13) to find the value of *L_B_*. 

### 2.4. The Linear Method 

The linear method for finding the overall binding rate, *r_B_*, uses the binding phenomenological Equation (1), which belongs to linear nonequilibrium thermodynamics [55]. Equation (1) combines *L_B_* with the Gibbs energy of binding, Δ*_B_G*. The value of Δ*_B_G* is calculated from the standard Gibbs energy of binding, Δ*_B_G*⁰, using the equation

(14) $${∆}_{B}G={∆}_{B}G^{0}+R_{g}T\ln Q$$

where *Q* is the reaction quotient [54,60,61]. *Q* is defined as the ratio of concentrations of reactants and the products of reaction (4) [54,60,61].

(15) $$Q=\frac{\left[AR\right]}{\left[A\right]\left[R\right]}$$

The calculation was made with *Q* = 0.91 *K_B_*. 

### 2.5. Exponential Method 

The exponential method is the third method used to find the overall binding rate, *r_B_*. It uses a more general exponential equation of nonequilibrium thermodynamics and is valid outside the linear region [54,55].

(16) $$r_{B}=r_{on}\left(1-e^{{∆}_{B}G/R_{g}T}\right)$$

This exponential equation can be used to derive the binding phenomenological Equation (1) [54,55]. When the values of the Gibbs energy of binding are small, the exponent can be approximated by a linear function: *e^x^* ≈ 1 + *x*, where *x* = Δ*_B_G*/*R_g_T* and *r_on_/R_g_* = *L_B_* [54,55]. 

## 3. Results

Standard Gibbs energies of binding were determined for BA.2.75, BA.2, BA.4/5, and other major SARS-CoV-2 variants. They are given in Table 1. Standard Gibbs energy of binding of BA.2 variant was found to be −45.81 kJ/mol, while for BA.5 it was −44.95 kJ/mol. Finally, for BA.2.75, the standard Gibbs energy of binding was found to be −49.91 kJ/mol.

Binding rates of the analyzed SARS-CoV-2 variants were calculated and are presented in Table 2. The binding rate for the BA.2 variant was found to be 6.58 × 10^−17^ M/s, while for BA.5 it was 1.19 × 10^−17^ M/s. Finally, for BA.2.75 it was 5.74 × 10^−18^ M/s, while for BA.2.75 (N460K) it was 1.49 × 10^−15^ M/s. 

The binding equilibrium constants of the analyzed SARS-CoV-2 variants were calculated and are shown in Table 1. The binding equilibrium constant of the BA.2 variant was found to be 1.06 × 10^8^ M/s, while for BA.5 it was 7.52 × 10^7^ M/s. The binding equilibrium constant of the BA.2.75 variant was 4.55 × 10^8^ M/s.

**Figure 1:**
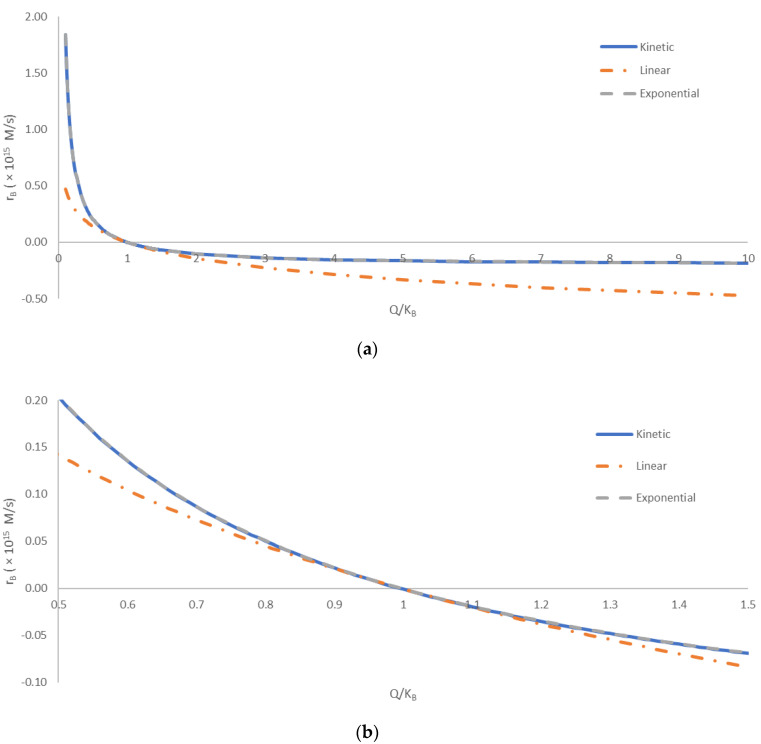
A comparison of the kinetic, linear, and exponential methods for calculating the overall binding rate, *r_B_*. The comparison was made for various distances from equilibrium, quantified by *Q*/*K_B_*. The equilibrium corresponds to *Q*/*K_B_* = 1. The region where *Q*/*K_B_* < 1 corresponds to the reaction being incomplete, with unreacted reactants that will form the product once the equilibrium is achieved. The region where *Q*/*K_B_* > 1 corresponds to the reaction “overshooting” the equilibrium, with excess product formed that will decompose into the reactants when the equilibrium is reached. (**a**) A comparison over a wide span of *Q*/*K_B_*, from 0.1 to 10. (**b**) Comparison close to equilibrium, with *Q*/*K_B_* between 0.5 and 1.5. The full blue line (**―**) represents the kinetic method, the orange dot-and-dash line (**- ∙ -**) represents the linear method, while the dashed gray line (**- - -**) represents the exponential method. The calculated overall binding rates have been multiplied by 10^15^ for clearer presentation.

Figure 1 shows a comparison of the kinetic, linear, and exponential methods for calculating the overall binding rate, *r_B_*. The three methods were compared at various distances from equilibrium. The distance from equilibrium was quantified by the ratio of the reaction quotient, *Q*, and the binding equilibrium constant, *K_B_*. The equilibrium corresponds to the point where *Q*/*K_B_* = 1. To the left, the region where *Q*/*K_B_* < 1 corresponds to the reaction being incomplete. In that region, according to Equation (15), there are excess reactant molecules that have not yet formed the product. To the right, the region where *Q*/*K_B_* > 1 corresponds to the reaction exceeding equilibrium. In this region, according to Equation (15), the product concentration is greater than that predicted by the equilibrium constant. Thus, the reaction will tend to flow in reverse, with a negative reaction rate, until the excess product dissociates into reactants. The comparison was made with *Q*/*K_B_* spanning two orders of magnitude. 

## 4. Discussion

The direction of the COVID-19 pandemic depends on two biological properties: the infectivity and pathogenicity of SARS-CoV-2 [50]. Infectivity and pathogenicity are biological properties, which are a consequence of virus–host interactions [49,68]. Virus–host interactions have a chemical and thermodynamic background [9,13,14,15,16,69,70,71,72]. Infectivity depends on the entry rate of the virus into susceptible cells [52]. Pathogenicity depends on the rate of virus multiplication [52]. Virus entry rate is a kinetic property. In its essence, the entry is preceded by antigen–receptor binding. Antigen–receptor binding represents a process similar to protein–ligand interactions [54,59]. The driving force for antigen–receptor binding is the Gibbs energy of binding [13,14,15,16,53]. Since 2019, SARS-CoV-2 has evolved continuously through the acquisition of multiple mutations [56]. According to the evolution theory, it is expected that mutations lead towards increases in infectivity and maintenance or decrease in pathogenicity [50]. Virus multiplication represents a chemical process of polymerization of nucleotides and amino acids into virus building blocks [70]. The driving force for virus population growth is the Gibbs energy of biosynthesis [50,54]. 

In this paper, the Gibbs energies of binding were calculated based on kinetic and thermodynamic properties, *k_on_*, *k_off_*, and *K_d_*, reported by Cao et al. [51] for the currently dominant BA.2.75 Omicron variant. The Gibbs energy of binding of the BA.2.75 Omicron variant was calculated to be −49.41 kJ/mol (Table 1). BA.2.75 is increasing in frequency, and had been detected in at least 15 countries as of the end of July 2022. This means that BA.2.75 is suppressing the existing BA.4 and BA.5 variants. This leads to the conclusion that infectivity of BA.2.75 is greater than that of BA.4 and BA.5. In that case, BA.2.75 is characterized by a more negative Gibbs energy of binding than BA.4 and BA.5. Moreover, the rate of entry into host cells depends on three factors: the Gibbs energy of binding, the binding phenomenological coefficient, and temperature. The temperature at which the most biological processes occur is the physiological temperature of 37 °C. The calculated binding phenomenological coefficients are given in Table 1. The calculated rates of binding of the viral spike glycoprotein trimer (SGP) to the human angiotensin-converting enzyme 2 (ACE2) are given in Table 2. Relative to the BA.2 variant, BA.2.75 carries nine additional mutations in the spike glycoprotein [73,74]. Mutation causes change in elemental composition and empirical formulae, leading to changes in thermodynamic properties. The underlying mechanism of BA.2.75’s enhanced infectivity, especially compared with BA.5, remains unclear for now [51].

Various Omicron strains compete for soil [50,72]. This means that BA.2.75 competes with BA.2 and BA.5. Since we know that BA.2.75 wins, it is expected to have a more negative Gibbs energy of binding than other variants, as well as greater entry rate and infectivity. Table 1 shows Δ*_B_G⁰* values for several SARS-CoV-2 variants. The Δ*_B_G*⁰ values of BA.2 and BA.5 variants were −45.81 kJ/mol and −44.95 kJ/mol, respectively. Indeed, Δ*_B_G⁰* of BA.2.75 is more negative than that of competing variants. This observation explains both the greater infectivity and suppression of previous variants by BA.2.75. 

The entry rate of SARS-CoV-2 variants was calculated using three approaches: kinetic, thermodynamic, and exponential. The kinetic approach uses the law of mass action with *k_on_* and *k_off_* rate constants [54,60,61]. The thermodynamic approach uses the binding phenomenological equation [54,55,75]. The exponential approach uses a more general exponential equation from nonequilibrium thermodynamics [54,55]. The results are shown in Table 2. The entry rates of BA.2 and BA.5 variants were found to be 6.58 × 10^−17^ M/s and 1.19 × 10^−17^ M/s, respectively. On the other hand, the entry rate of BA.2.75 was found to be 5.74 × 10^−18^ M/s using the kinetic method. This can be explained by a difference in binding phenomenological coefficients, *L_B_*. However, the variant BA.2.75 (N460K) exhibited the greatest binding rate of 1.49 × 10^−15^ M/s. Thus, the binding rate of BA.2.75 (N460K) is 23 times greater than that of BA.2 and 125 times greater than that of BA.5. 

The greater binding rate of BA.2.75 is in agreement with the constructal law. The constructal law states that “for a finite-size flow system to persist in time (to live) it must evolve such that it provides greater and greater access to the currents that flow through it” [36,37,38]. From the perspective of nonequilibrium thermodynamics, the binding rate is a flow driven by the Gibbs energy of binding [55,75]. Moreover, the binding rate was found to become greater during evolution of SARS-CoV-2 variants, from BA.2, through BA.5, to BA.2.75. Thus, the virus population as a system gradually through evolution increased the binding rate as a flow. 

The predictions of the kinetic, linear, and exponential methods are compared in Figure 1. The kinetic and exponential methods provided very similar results throughout the entire tested range of *Q*/*K_B_*. The difference in the predicted *r_B_* values of the kinetic and exponential methods was below 1% for most of the analyzed *Q*/*K_B_* range. The only exception is the area close to equilibrium, with *Q*/*K_B_* values from 0.85 to 1.21, where the relative discrepancy was greater, due to small values of *r_B_*. The good agreement of the kinetic method and exponential method indicates that nonequilibrium thermodynamics can provide accurate predictions of rates of biological processes, based on thermodynamic properties. On the other hand, the linear method deviated more from the kinetic and exponential methods. The deviation was the smallest in the area close the equilibrium value of *Q*/*K_B_* = 1, being less than 10% for *Q*/*K_B_* values from 0.80 to 1.21. This can be explained by the assumption made in the linear method, i.e., that the driving force Δ*_B_G* is not high [55]. In the area close to equilibrium, the Δ*_B_G* was small (close to zero), making the linear method the most accurate. However, all three methods showed the same general trend throughout the analyzed range of *Q*/*K_B_* values. Thus, the simplicity of the linear method and its connection of thermodynamics and kinetics still make it a valuable tool in thermodynamic analysis of biological phenomena. This is in agreement with the results of refs. [4,55,76,77], who found that the linear method can accurately predict multiplication rates of microorganisms. 

The mutations S446G and N460K are present in the BA.2.75 variant. They were found to provide the BA.2.75 variant enhanced resistance to neutralizing antibodies [78]. However, it seems that it is not only the evasion of the immune response, but also a more negative Gibbs energy of binding and entry rate into host cells, as shown by results in Table 2.

SARS-CoV-2 appeared in 2019 as the Hu-1 variant (wild type), causing the COVID-19 pandemic [79,80]. Since 2019, the virus has mutated several dozen times [81]. During mutations, new variants appeared, with different infectivity, pathogenicity, chemical compositions, and thermodynamic properties, causing pandemic waves. Evolution theory predicts that some of the mutations in the virus will lead to increases in infectivity. Increases in infectivity during the competition of variants circulating in the population lead to the suppression of older variants and the domination of newer ones. Thus, appearance of new variants has caused an intense effort of the scientific community on characterization and assessment of danger to human population, while, on the other hand, we have often encountered panicked, inaccurate predictions by the media and general population. In this paper, an assessment of infectivity was performed based on a mechanistic model, used for other SARS-CoV-2 variants, just a few days after the publication of data on kinetics of antigen–receptor interactions for BA.2.75. BA.2.75 possesses nine new mutations compared with earlier variants. These mutations have led to a change in chemical composition and binding affinity. These changes have led to changes in thermodynamic properties of binding, which lead to changes in the antigen–receptor binding rate. These changes in the binding rate lead to changes in the infectivity of the new variant. The results of this research showed that the Gibbs energy of binding of BA.2.75 was more negative than that of BA.2 and BA.5. Moreover, the rate of antigen–receptor binding was greater for BA.2.75. Thus, the BA.2.75 subvariant exhibited greater infectivity. It seems that the evasion of immune responses is not the only mechanism that leads to the suppression of older variants by BA.2.75. 

## 5. Conclusions

The Gibbs energy of binding of the Omicron BA.2.75 subvariant is more negative than that of the competing BA.2 and BA.5 variants. This may be the reason why the BA.2.75 subvariant has exhibited a high infectivity in India and other countries. 

Mutation N460K on the BA.2.75 subvariant contributes not only to evading the immune response, but also to faster antigen–receptor binding. Thus, the infectivity of this subvariant is greater than that of competing variants. 

The greatest rate of binding to host-cell receptors is that of BA.2.75 with the mutation N460K, being 23 times greater than that of BA.2 and 125 times greater than that of BA.5.

The greater binding rate gives an advantage to the BA.2.75 subvariant compared with BA.2 and BA.5 during competition between the variants circulating in the population. Thus, BA.2.75 can suppress BA.2 and BA.5, leading to the development of a new pandemic wave. 

## Figures and Tables

**Table 1:** Standard thermodynamic properties of the binding of SARS-CoV-2 variants. The table shows the association rate constant, *k_on_*, dissociation rate constant, *k_off_*, dissociation equilibrium constant, *K_d_*, binding phenomenological coefficient, *L_B_*, binding equilibrium constant, *K_B_*, and standard Gibbs energy of binding, Δ*_B_G*⁰; data taken at 25 °C. The *k_on_*, *k_off_*, and *K_d_* data were taken from ref. [51].

Name	*k_on_* (M^−1^s^−1^)	*k_off_* (s^−1^)	*K_d_* (M)	*L_B_* (mol^2^ K/J s dm^3^)	*K_B_* (M^−1^)	Δ*_B_G*⁰ (kJ/mol)
BA.2	4.06 × 10^6^	3.82 × 10^−2^	9.40 × 10^−9^	8.01 × 10^−17^	1.06 × 10^8^	−45.81
BA.4/5	5.30 × 10^5^	7.07 × 10^−3^	1.33 × 10^−8^	1.48 × 10^−17^	7.52 × 10^7^	−44.95
BA.2.75	1.88 × 10^6^	4.22 × 10^−3^	2.20 × 10^−9^	8.68 × 10^−18^	4.55 × 10^8^	−49.41
BA.2.75 (Q493R)	8.85 × 10^5^	5.64 × 10^−3^	6.40 × 10^−9^	1.19 × 10^−17^	1.56 × 10^8^	−46.77
BA.2.75 (S446G)	3.36 × 10^6^	1.18 × 10^−2^	3.50 × 10^−9^	2.47 × 10^−17^	2.86 × 10^8^	−48.26
BA.2.75 (N460K)	3.87 × 10^7^	5.02 × 10^−1^	1.38 × 10^−8^	1.12 × 10^−15^	7.25 × 10^7^	−44.86
B.1.1.7 (Alpha)	7.38 × 10^5^	3.55 × 10^−3^	4.80 × 10^−9^	7.43 × 10^−18^	2.08 × 10^8^	−47.48
B.1.351 (Beta)	5.42 × 10^5^	7.31 × 10^−3^	1.35 × 10^−8^	1.54 × 10^−17^	7.41 × 10^7^	−44.92
P.1 (Gamma)	3.77 × 10^5^	6.29 × 10^−3^	1.67 × 10^−8^	1.32 × 10^−17^	5.99 × 10^7^	−44.39
B.1.617.2 (Delta)	7.21 × 10^5^	7.84 × 10^−3^	1.09 × 10^−8^	1.65 × 10^−17^	9.17 × 10^7^	−45.45
BA.1	1.04 × 10^6^	1.07 × 10^−2^	1.03 × 10^−8^	2.25 × 10^−17^	9.71 × 10^7^	−45.59
BA.2.12.1	9.08 × 10^5^	9.41 × 10^−3^	1.04 × 10^−8^	1.98 × 10^−17^	9.62 × 10^7^	−45.56
BA.3	1.54 × 10^6^	3.16 × 10^−2^	2.04 × 10^−8^	6.59 × 10^−17^	4.90 × 10^7^	−43.89
BA.2.75 (H339)	2.81 × 10^6^	6.72 × 10^−3^	2.40 × 10^−9^	1.41 × 10^−17^	4.17 × 10^8^	−49.20

**Table 2:** The binding rates of SARS-CoV-2 variants. The table shows *r_kin_*, *r_TD_*, and *r_exp_*: binding rates calculated using the kinetic, thermodynamic, and exponential methods, respectively. The values were calculated at *Q* = 0.91 *K_B_*.

Name	*r_kin_* (M/s)	*r_TD_* (M/s)	*r_exp_* (M/s)
BA.2	6.58 × 10^−17^	6.34 × 10^−17^	6.64 × 10^−17^
BA.4/5	1.19 × 10^−17^	1.17 × 10^−17^	1.23 × 10^−17^
BA.2.75	5.74 × 10^−18^	6.88 × 10^−18^	7.20 × 10^−18^
BA.2.75 (Q493R)	1.03 × 10^−17^	9.42 × 10^−18^	9.86 × 10^−18^
BA.2.75 (S446G)	1.98 × 10^−17^	1.95 × 10^−17^	2.05 × 10^−17^
BA.2.75 (N460K)	1.49 × 10^−15^	8.88 × 10^−16^	9.29 × 10^−16^
B.1.1.7 (Alpha)	6.03 × 10^−18^	5.89 × 10^−18^	6.16 × 10^−18^
B.1.351 (Beta)	1.29 × 10^−17^	1.22 × 10^−17^	1.27 × 10^−17^
P.1 (Gamma)	1.11 × 10^−17^	1.05 × 10^−17^	1.10 × 10^−17^
B.1.617.2 (Delta)	1.40 × 10^−17^	1.31 × 10^−17^	1.37 × 10^−17^
BA.1	1.88 × 10^−17^	1.78 × 10^−17^	1.86 × 10^−17^
BA.2.12.1	1.70 × 10^−17^	1.57 × 10^−17^	1.64 × 10^−17^
BA.3	5.15 × 10^−17^	5.22 × 10^−17^	5.47 × 10^−17^
BA.2.75 (H339)	1.22 × 10^−17^	1.12 × 10^−17^	1.17 × 10^−17^
